# Enhanced Thermal Stability of Thermoplastic Polymer Nanostructures for Nanoimprint Lithography

**DOI:** 10.3390/ma12030545

**Published:** 2019-02-12

**Authors:** Youwei Jiang, Bingqing Luo, Xing Cheng

**Affiliations:** 1 SUSTech Academy for Advanced Interdisciplinary Studies, Southern University of Science and Technology, Shenzhen 518055, China; 2 Shenzhen Key Laboratory for Nanoimprint Technology, Southern University of Science and Technology, Shenzhen 518055, China; luobq@sustc.edu.cn; 3 School of Innovation and Entrepreneurship, Southern University of Science and Technology, Shenzhen 518055, China; 4 Department of Materials Science and Engineering, Southern University of Science and Technology, Shenzhen 518055, China

**Keywords:** thermal nanoimprint, polymer patterning, polymer reflow, step-and-repeat nanoimprint

## Abstract

Thermoplastic polymer micro- and nanostructures suffer pattern decay when heated to a temperature close to or above the polymer’s glass transition temperature. In this work, we report enhanced thermal stability of polycarbonate nanostructures at temperatures well above their glass transition temperatures. Based on this observation, we develop a unique technique for high-resolution polymer patterning by polymer reflows. This technique is characterized as the precise control of polymer reflows regardless of the annealing time, which avoids the time-domain nonlinear reflow of the polymer melt. We also implement thermal nanoimprinting in a step-and-repeat fashion, which dramatically increases the throughput of the thermal nanoimprint. The enhanced pattern stability against thermal reflow also allows for multiple imprinting at the same location to generate complex resist patterns from a simple mold structure. Since modern lithography often uses thin resist films (sub-100 nm) due to the restraint from the pattern aspect ratio, the unusual annealing behavior of thin polymer films is highly relevant in sub-100 nm lithographic processing.

## 1. Introduction

Nanoimprint lithography (NIL) is a high throughput and low cost patterning technique which has been developed to exceed the resolution limit of photolithography [1,2,3,4,5,6,7,8,9,10,11]. NIL forms nanoscale patterns in a thermoplastic polymer film by deforming the polymer under high pressure at an elevated temperature with a hard mold. In recent years, both UV [9,12,13,14,15,16,17,18,19,20] and thermal [6,15,21,22,23,24] nanoimprints have demonstrated sub-10 nm resolution. At the same time, other lithography techniques with high resolution, or those with large area and low cost patterning capability, have drawn a lot of attentions. For example, electron-beam lithography (EBL) could achieve ultra-high resolution as good as 2 nm [25,26,27]. However the throughput of EBL is relatively limited. Block copolymer lithography [28,29,30,31] and nanosphere lithography [32,33] rely on the copolymer phase separation or particle self-assembly to form large-area nanostructures without complicated instrumentation. Laser interference lithography is a maskless patterning technique which could provide large-scale nanolithography with coherent laser beams [34,35,36]. Nanoimprint lithography is characterized as high resolution, high throughput, and low cost. However, there remain various issues in NIL before the acceptance of imprint-based technologies in industrial applications. For thermal NIL, a critical issue in terms of pattern stability is related to internal stress relaxation at a temperature close to or even below the glass transition temperature (T_g_) of the polymer after imprinting [37,38,39,40]. This issue also limits the throughput for thermal NIL, due to a necessary cooling process prior to mold release [11,38,41,42]. At the same time, UV nanoimprinting is more suited for large scale commercial applications, since it can be operated in a step-and-flash fashion [19,43].

The reflow behavior of polymer micro- and nanostructures fabricated by thermal NIL has been widely investigated [44,45,46,47,48,49]. In general, pattern relaxation can be observed by thermal annealing of the polymer patterns at temperatures above T_g_, and with extended annealing time the pattern shape is no longer maintained due to excessive polymer melt flow. In this work, we investigated the reflow behavior of polycarbonate nanostructures patterned by nanoimprinting. We found that the reflow behavior of thermoplastic polycarbonate nanostructures exhibits strong dependency on pattern heights. For nanostructures with relatively large heights, although pattern relaxation was observed at the initial stage of the thermal annealing process, stable patterns formed after reaching the “end point” of the reflow, which relied on the initial thickness of the patterns prior to thermal annealing. On the other hand, shallow nanostructures of polycarbonate showed enhanced stability upon thermal annealing at temperatures well above the T_g_ of the polycarbonate. 

A potential application of our finding is high-resolution surface patterning by well-controlled thermal reflow. A minimum trench size of ca. 35 nm was achieved with starting patterns of 700 nm pitch (50% duty cycle) gratings. We also developed a facile step-and-repeat thermal nanoimprint technique, which is so far difficult to achieve, by taking advantage of this unexpected phenomenon.

## 2. Experimental Section

**Figure 1:**
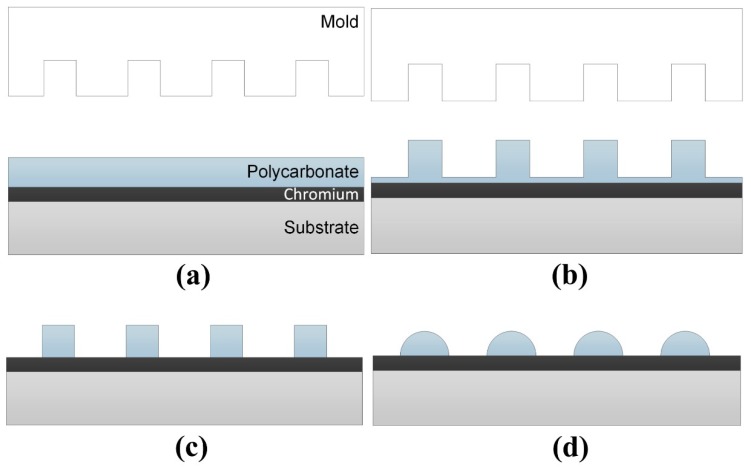
A schematic of polycarbonate nanoimprint and thermal annealing: (**a**) Polycarbonate resist on a chromium layer; (**b**) thermal nanoimprint to form polycarbonate nanostructures; (**c**) oxygen reactive-ion etching (RIE) to remove the residual layer after nanoimprinting; (**d**) annealing of polycarbonate nanostructures on a hot plate at elevated temperatures to induce polymer reflow.

Polycarbonate patterns studied in this work were fabricated by thermal nanoimprinting. After nanoimprinting, we used oxygen reactive-ion etching to fine control the heights of the patterns. The polycarbonate nanostructures with various pattern heights were than annealed at elevated temperatures. The final patterns after annealing were analyzed. The procedure is shown in Figure 1. 

### 2.1. Nanoimprint Mold Fabrication

Two types of nanoimprint molds were prepared. A Si mold with an array of circular holes (50 nm diameter, 100 nm pitch, and 100 nm depth) was used to fabricate polycarbonate pillars at the sub-50 nm scale. A 700 nm period SiO_2_ mold with 50% duty cycle and 250 nm depth was used to carry out a series of experiments to investigate the end point of polymer reflows with respect to the initial thickness of the patterns. Those two molds were fabricated by electron-beam lithography and dry etching. All molds were coated with a monolayer of 1H,1H,2H,2H-perfluorodecyltrichlorosilane (FDTS, from Gelest) as a hydrophobic surfactant layer for easy mold release. The molds were soaked in FDTS solution in heptane (0.1 mL FDTS dispersed in 100 mL heptane) for 10 min. After pure heptane rinsing, the molds were baked on a 110 °C hot plate for 2 min.

### 2.2. Polycarbonate Thin Films 

The polymer resist used in this work is polycarbonate resin (secondary standard, Scientific Polymer Product, Inc., Ontario, NY, USA) with an average molecular weight of 36.6 kg/mol. Polycarbonate solutions of 1 and 4 wt% concentrations were prepared by dissolving polycarbonate powders in cyclohexane solvent. Polycarbonate thin films were obtained by spin-coating the solutions on a SiO_2_ layer or a 40 nm chromium layer which was thermally evaporated onto a Si substrate. After spin-coating, the residual solvent in polycarbonate thin film was removed by baking the substrates on a 100 °C hotplate for 3 min. The 1 wt% solution was used to prepare a relatively thin film and a 20 nm polycarbonate film was prepared by spin-coating the 1 wt% polycarbonate in cyclohexane solution at 4000 rpm for 1 min. Meanwhile, the 4 wt% solution was used for relatively thick film preparation and a 130 nm polycarbonate film was prepared by spin-coating the 4 wt% polycarbonate in cyclohexane solution at 4000 rpm for 1 min. The thickness of the polycarbonate thin films was measured by an ellipsometer (Nanofilm EP3-SE, Accurion, Germany).

### 2.3. Polycarbonate Nanoimprint and Thermal Annealing

Polycarbonate resist (T_g_ = 154 °C) was imprinted by Si molds with holes of 50 nm in diameter and 100 nm in depth at 220 °C and 5 MPa. Oxygen reactive-ion etching (RIE, NAURA GSE-200, Beijing, China) was applied to remove the residual layer after imprinting. The etching rate for the polycarbonate residue layer removal process is around 65 nm/min, with parameters of 20 sccm O_2_, 50 W RF power, and 10 mTorr chamber pressure. Over-etching of the residual layer was intentionally employed to control the final height of the pillar patterns to investigate the impact of pattern height on annealing behaviors. The heights of the polycarbonate patterns were measured by atomic force microscopy (AFM) scan (Dimension Icon, Bruker, Billerica, MA, USA). The polycarbonate patterns were annealed at elevated temperatures from 154 to 300 °C, which is well above the T_g_, for various time periods. The pattern morphologies after thermal annealing were characterized by scanning electron microscopy (JSM-7500F, JEOL, Tokyo, Japan). The SEM images of the polycarbonate nanostructures before and after annealing were analyzed to obtain the distribution of pattern sizes. The statistical data were used in studying the reflow behavior of the polycarbonate nanostructures.

### 2.4. Polycarbonate Grating Reflow and End-Point Control

As a potential application of the thermal stability of polycarbonate nanostructures, we fabricated polycarbonate gratings and performed reflow to achieve structures with much narrower trench patterns than the original grating structures. To test the feasibility of this technique, a polycarbonate film of 130 nm was prepared by spin-coating polycarbonate solutions on a SiO_2_ layer. Then, the polycarbonate resist was imprinted by the 700 nm period SiO_2_ mold (50% duty cycle) with 250 nm depth. The residual layer was removed and the final height of the gratings was controlled by oxygen RIE time. Then, thermal annealing was carried out at 200 °C for 30 min. The pattern morphology was characterized by JEOL JSM-7500F SEM.

## 3. Results and Discussion

### 3.1. Enhanced Thermal Stability of Polycarbonate Patterns

**Figure 2:**
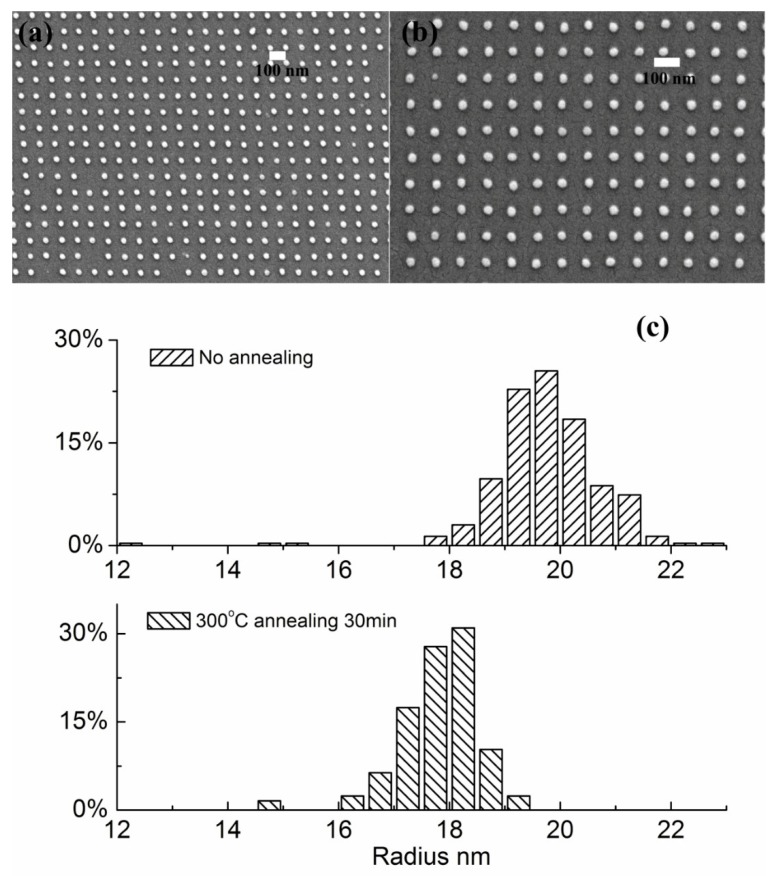
The SEM images of the polycarbonate pillars (**a**) before and (**b**) after thermal annealing, and (**c**) the distribution of the radius of the polycarbonate pillars before and after thermal annealing.

In order to investigate the thermal stability of sub-50 nm polycarbonate pillars, we annealed the nanostructures at 300 °C for 5, 10, 20, and 30 min. The SEM images for the polycarbonate nanostructures before and after 30 min annealing at 300 °C are shown in Figure 2a,b, respectively. The distribution of the radii of the polycarbonate pillars was plotted in Figure 2c. The heights of the polycarbonate pillars were controlled to be around 35 nm. Instead of spreading on the substrate surface, the polycarbonate pillars were stable and the average radius of the post-annealing pillars reduced to 18 nm from 19–20 nm. The reduction of the average radius after annealing clearly demonstrated the enhanced thermal stability of the polycarbonate nanostructures. Although only the diameter distribution information for samples annealed for 30 min is presented here, it was observed that all samples annealed for 5, 10, 20, and 30 min were identical, indicating the rapid formation of the final structures in the first few minutes and no flow of polymer melt in the remaining annealing period. 

The enhanced thermal stability of polycarbonate patterns can be ascribed to the chain entanglement in polymer thin films. In the tube model [50] for the entangled polymers, the motion of a single polymer chain is suppressed by the neighboring chains within a tube-like region, and polymer chains with molecular weight higher than M_e_, the entanglement molecular weight, will be dramatically affected by the constraints within the tube. Wu [51] and Fetters [52] et al. reported the M_e_ of polycarbonate to be 1.78 and 1.33 kg/mol, respectively. For polycarbonate (M_w_ = 36.6 kg/mol) used in this work, the molecular weight is equivalent to 20.56 to 27.52 M_e_, which indicates a considerable chain entanglement and chain motion suppression. In this case, the wetting behavior [53,54,55] of entangled polymer thin films could be different from those without entanglement. If the polymer chain is in the entanglement region, the energy required to stretch the chains will be dominating and total wetting is unlikely to happen. Zhao [56] et al. reported the wetting properties of liquid polyethylene propylene (PEP) on native SiO_2_ and HF-etched Si substrate. They observed that dewetting occurs when the PEP film thickness is smaller than its radius of gyration (R_g_). By considering the energy of stretching the chains, they theoretically estimated the minimum film thickness required for total wetting. A similar explanation can be applied to our observations. The radius of gyration of polycarbonate can be estimated by [57] $R_{g}=0.038\sqrt{M_{w}}$, which is 7.3 nm for polycarbonate of molecular weight 36.6 kg/mol. If the height of polycarbonate structures is much greater than the R_g_, spreading of polymer melt can be observed due to thermal reflow. However, when the pattern height is reduced to just about 6–8 times of its R_g_, the pattern no longer reflows due to strong internal chain entanglement. 

The extensive cross-linking of the polycarbonate thin film surface caused by oxygen RIE could be another contributing factor for the pattern stability. Larsson et al. [58] reported increased solvent, storage, thermal stability, and higher Young’s modulus of polycarbonate surfaces treated by oxygen plasma with high self-bias voltages. Sharma et al. [59] reported a decrease in the O:C ratio of polycarbonate surfaces with a prolonged oxygen plasma treatment, which indicated an increasing surface cross-linking. In this work, oxygen RIE is applied to remove the residual layer and control the thickness of the patterns, and therefore the change of the thin film properties could lead to the enhanced thermal stability observed in this work. However, the impact of plasma treatment is not well understood yet in this work. More analysis regarding the detailed reaction mechanism, such as nano-indentaion, X-ray photoelectron spectroscopy (XPS), and rheology are necessary to gain insight towards this phenomenon.

### 3.2. Reflow of Polycarbonate Gratings and Its Application in Nanopatterning

We also investigated the reflow behavior of polycarbonate gratings with pattern height above 50 nm. Although the patterns spread on the substrate at the early stage by thermal annealing, they finally ended up with stable structures without further reflow, regardless of the annealing time. The stable pattern is achieved when the thickness of the front end of the polymer melt is reduced to around 6–8 R_g_ due to the spreading of the polymer melt. This feature can be utilized for nanopatterning by a precise control of the polymer reflow. The final feature size after the reflow can be determined by the initial height of the polycarbonate gratings, which in turn can be precisely controlled by oxygen plasma etching recipe and etching time. 

**Figure 3:**
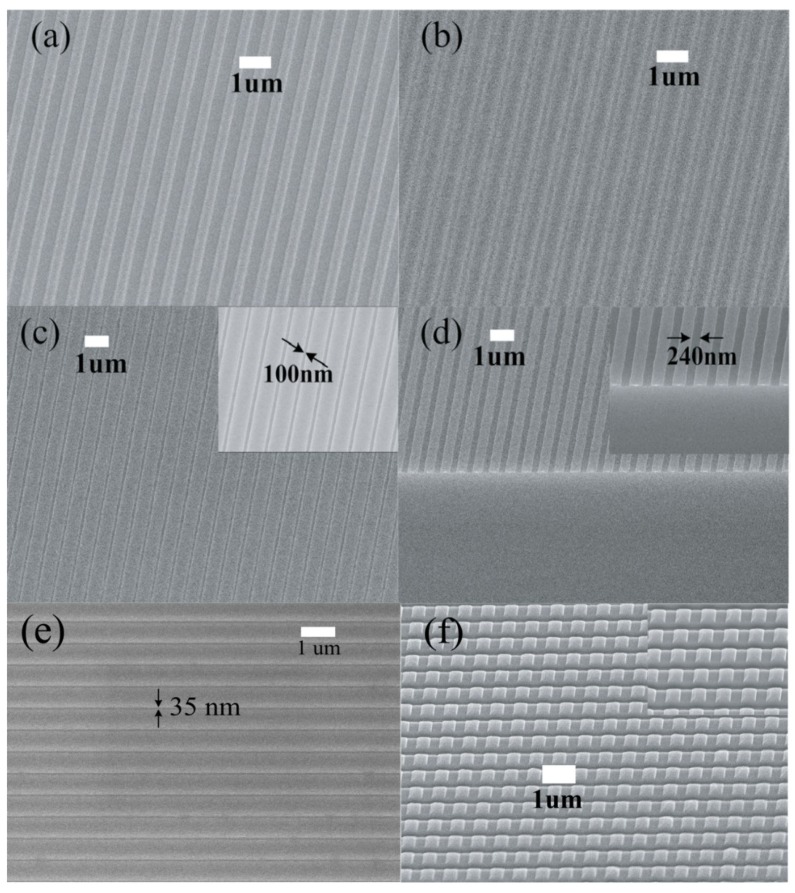
(**a**) Polycarbonate gratings with 150 nm height fabricated by nanoimprint and RIE, (**b**) polycarbonate patterns formed by annealing the gratings in (**a**) at 200 °C for 30 min, (**c**) SiO_2_ gratings with 100 nm trenches fabricated by CHF_3_ RIE with polycarbonate as etching masks from (**b**), (**d**) SiO_2_ gratings with 240 nm trenches fabricated by CHF_3_ RIE with polycarbonate as etching masks, annealed from 70 nm thick polycarbonate gratings, (**e**) polycarbonate gratings with 35 nm trench size after thermal annealing, and (**f**) dual imprints of 90 nm thick polycarbonate film.

The polycarbonate gratings were fabricated by thermal nanoimprinting and the heights of the patterns were controlled to be 150 (Figure 3a) and 70 nm by oxygen RIE. Samples were placed on a hotplate at 200 °C for 30 min to allow polymer reflow and the formation of stable structures. The shrinking of the grating trenches after the thermal annealing can be observed in Figure 3a,b. The annealed polymer grating features can be further transferred into a SiO_2_ layer by CHF_3_ RIE. The etching rate for SiO_2_ etching is around 13 nm/min, with parameters of 30 sccm CHF_3_, 5 sccm O_2_, 200 W RF power, and 10 mTorr chamber pressure. SiO_2_ gratings with 100 nm trench size in Figure 3c were obtained by RIE pattern transfer from Figure 3b. In Figure 3d, SiO_2_ gratings with 240 nm trench size were achieved from the annealed polycarbonate patterns with 70 nm initial thickness.

**Figure 4:**
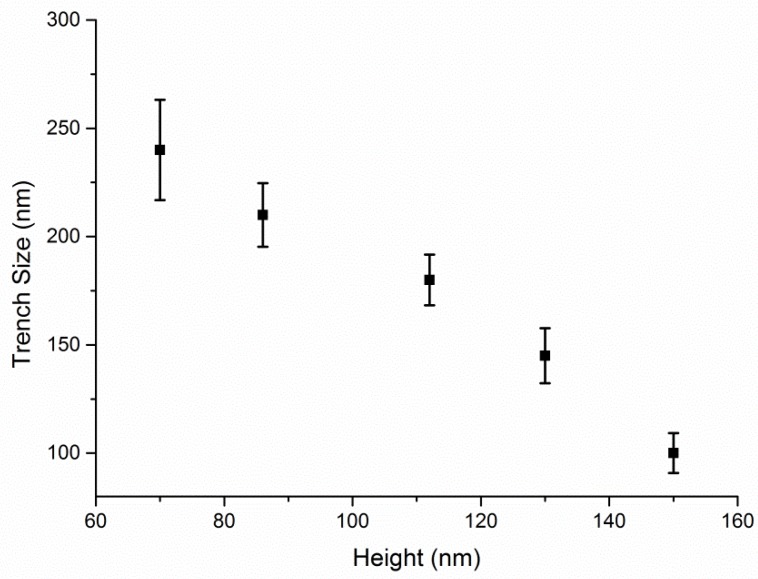
The widths of the trenches transferred onto an oxide layer by dry etching as a function of the initial polycarbonate pattern heights.

The trench width after thermal annealing depends on the initial thickness of the polycarbonate gratings, which can be controlled by the oxygen RIE time. Smaller trench size is expected from thicker gratings due to more extended reflow of the polymer melt. However, partial or complete merging of adjacent patterns can happen if the gratings are too high. We designed a series of experiments to investigate the dependency of final trench width on initial grating height. Figure 4 shows how the widths of the trenches transferred into an oxide layer by dry etching are related to the initial thicknesses of the grating heights. Based on this observation, larger initial pattern height will result in smaller trench widths and this could be employed for high-resolution polymer patterning. Figure 3e shows that a minimum trench width of 35.0 ± 5.2 nm was achieved from a 350 nm wide trench with 200 nm height, which is a 90% reduction of the original pattern size. In microelectronic processing, film thickness is easy to be precisely controlled. Thus, here we enable a facile approach to achieve precise high-resolution nanostructures without advanced lithographic techniques. 

The capability of multiple imprints at the same location can also be realized due to the thermal stability of the polymer patterns. Experimentally, a polycarbonate film of 90 nm in thickness was imprinted by the same grating mold used previously at 200 °C and 5 MPa. After 5 min pressing, the mold was released from the substrate without cooling and placed immediately on top of the patterned region for the second imprint. The nanoimprint mold was aligned in the orthogonal direction and the imprinting procedure was the same with the first imprint. Figure 3f shows the grid structures patterned by dual imprints with the grating mold. Instead of being destroyed by the heat and pressure during the second imprint, the imprinted polycarbonate gratings in first imprint remained stable after achieving the “end point” of thermal reflow, and the final checkbox patterns were produced by sequential nanoimprints with a grating mold. Thus, the thermal stability of very thin polymer films enables the patterning of complex polymer structures from molds with simpler patterns by sequential nanoimprints at the same location.

### 3.3. Step-and-Repeat Thermal Nanoimprinting

Lithography is commonly done in a step-and-repeat fashion so that patterns on smaller molds (or masks) can be replicated on a much larger substrate, particularly in commercial manufacturing. Although step-and-flash UV nanoimprinting has already been developed long ago, thermal nanoimprinting in a step-and-repeat fashion has not been widely used due to the difficulty to implement. This is because the polymer micro- and nanostructures patterned in previous imprinting steps will reflow and collapse during later imprinting steps when the substrate is kept at the imprinting temperature. Gourgon et al. [60] analyzed sequentially imprinted polymer gratings with scatterometry and reported pattern reflow due to heat diffusion at various temperatures. Local heating of the mold or temperature zoning of the substrate may address the issue. Yoon et al. [61] reported a step-and-repeat thermal NIL system, which consisted of a metal screen to achieve selective heating of the resist and a copper heat sink to prevent lateral heat conduction. However, this method increases tool complexity and narrows the processing window. Francone et al. [62] and Haatainen et al. [63] reported a step-and-repeat thermal NIL process by local heating of the mold. However, a repetitive heating and cooling cycle of the mold is still inevitable, which limits the throughput of the process.

**Figure 5:**
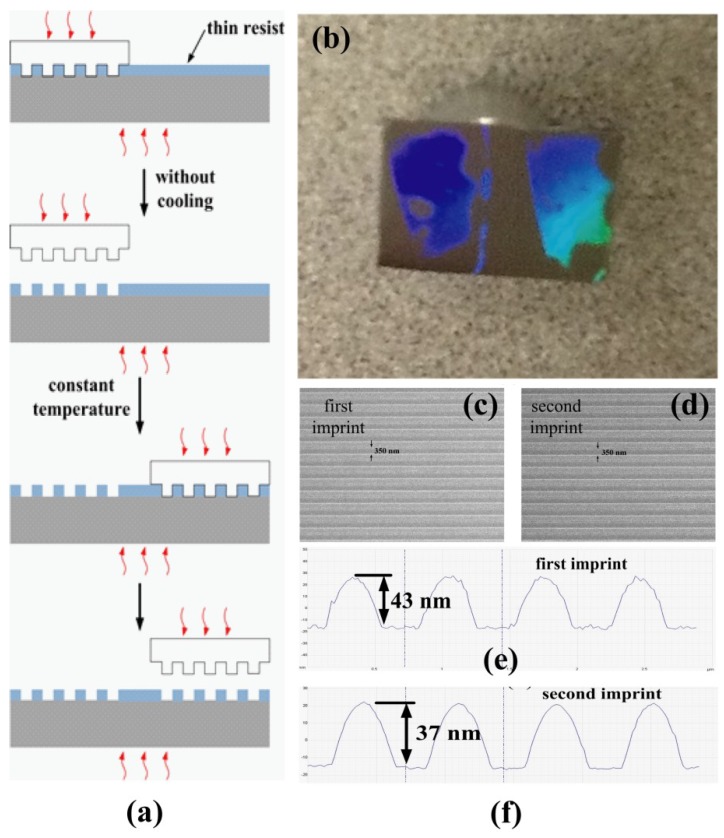
(**a**) The schematics of step-and-repeat thermal nanoimprinting and (**b**) a photograph of two imprinted regions within the same substrate by a step-and-repeat thermal nanoimprinting. (**c**,**d**) are SEM images of polycarbonate gratings patterned by the first imprint and the second imprint, respectively. (**e**,**f**) are the heights of the gratings of the first and the second imprint scanned by AFM, respectively.

Here we take advantage of the enhanced stability of polycarbonate nanostructures to implement a step-and-repeat thermal nanoimprint, and Figure 5a shows the schematics of this technique. Experimentally, a polycarbonate film of around 20 nm thickness was prepared by spin-coating 1 wt% polycarbonate solution in cyclohexane at 4000 rpm for 1 min. Residual solvent was removed by 100 °C baking on a hotplate for 3 min. After that, the first imprint was operated at 200 °C, 5 MPa for 5 min. Then, the mold (700 nm pitch, 50% duty cycle, and 250 nm depth) was released immediately without cooling and moved to the adjacent region within the same substrate for the second imprint. The temperature of the mold–substrate stack was maintained at 200 °C until the sample was removed from the hot press after the second imprint. Figure 5b–f shows the results of two imprints on the same substrate by the step-and-repeat thermal nanoimprinting. In Figure 5b, the photograph demonstrated the coexistence of two imprinted regions, corroborating the enhanced thermal stability of polycarbonate structures patterned during the imprints. The polycarbonate gratings were characterized by SEM and the corresponding images are shown in Figure 5c,d. No obvious pattern decay or reflow can be observed from the patterns formed in the first and second imprints. Defects are visible within the imprinted area in Figure 5b, since the mold–substrate stack was pressed between metal platens in an ambient environment in our experiment, and the contact between the mold and the substrate was not uniform due to the limitation of the hot press. The sidewalls for polycarbonate gratings were not vertical, as shown in the AFM scans (Figure 5e,f), since the mold trench was much deeper than the film thickness. This could be improved by using a shallower mold with around 40 nm trench depth.

## 4. Conclusions

In summary, we observed the enhanced thermal stability of polycarbonate nanostructures at temperatures well above their glass transition temperature. The stability against thermal annealing occurs in polycarbonate nanostructures with a pattern height of around 6 to 8 times of the radius of gyration of the polymer chain. The stability could be a result of strong chain entanglement, because the benzene rings on the polycarbonate main chains easily interlock with each other to form strong entanglement that is not easy to be unraveled. The increased surface cross-linking could be another contributing factor towards the enhanced stability. However, the detailed mechanism is not yet clear in this work. The unexpected thermal stability can be utilized in many ways. First, it can be used to precisely determine the “end point” of thermal reflow of polymer patterns, and this feature can be utilized to deterministically control the final dimension of polymer structures in the thermal reflow process. We achieved a trench width of 35 nm from an original 350 nm trench by the precise thermal reflow process. Second, the enhanced thermal stability of polycarbonate nanostructures enables facile step-and-repeat thermal nanoimprinting, which is otherwise difficult to implement. This opens many possibilities in thermal nanoimprinting, such as patterning a large substrate from a small template, and significantly increase the throughput of thermal nanoimprinting because temperature cycling can be eliminated in conventional thermal nanoimprinting. Moreover, it enables multiple thermal nanoimprints at the same area to form a complex pattern from molds of simpler patterns. Despite the limitation of well-entangled materials, these techniques may still find promising applications for large-area and complex nanopatterning by thermal nanoimprinting. Finally, the polymer resists used in modern day lithographic techniques are on the order of tens of nanometers, which is in the same thickness range as the polycarbonate nanostructures studied here. The findings of this work may be relevant to other thin polymer films used in advanced lithographic techniques, and the enhanced thermal stability of polymer nanostructures may enable practices for unconventional patterning applications.
